# Intramural duodenal hematoma: an unusual complication after esophagogastroduodenoscopy in an adolescent

**DOI:** 10.1055/a-1838-4386

**Published:** 2022-05-25

**Authors:** Paolo Cocco, Enrico La Pergola, Cosimo Bleve, Lorenzo Costa, Alessandro Brandolese, Nicola Schiavone, Salvatore Fabio Chiarenza

**Affiliations:** 1 Department of Pediatric Surgery and Pediatric Minimally Invasive Surgery and New Technologies, San Bortolo Hospital, Vicenza, Italy; 2 Gastroenterology and Digestive Endoscopy Unit, San Bortolo Hospital, Vicenza, Italy

Intramural duodenal hematoma (IDH) is a rare complication after small-bowel endoscopic biopsy; it occurs mainly in children with impaired coagulation 1 2 3 4 5 . We report the case of a 13-year-old girl with no relevant medical history who was complaining of abdominal tenderness and failure to thrive and underwent a diagnostic esophagogastroduodenoscopy (EGD) with duodenal biopsies ( Fig. 1 ). After 24 hours, she presented with nonbloody, nonbilious vomiting, and diffuse and intense abdominal pain. Abdominal ultrasound and a contrast-enhanced computed tomography scan revealed an asymmetric thickening of the duodenal wall measuring 80 × 50 mm, suggestive of an IDH ( Fig. 2 ). Laboratory testing showed that she had previously unknown platelet dysfunction. Only at this time did the family reveal details of an episode of hemarthrosis after minor trauma that they had previously omitted to mention.

**Fig. 1:**
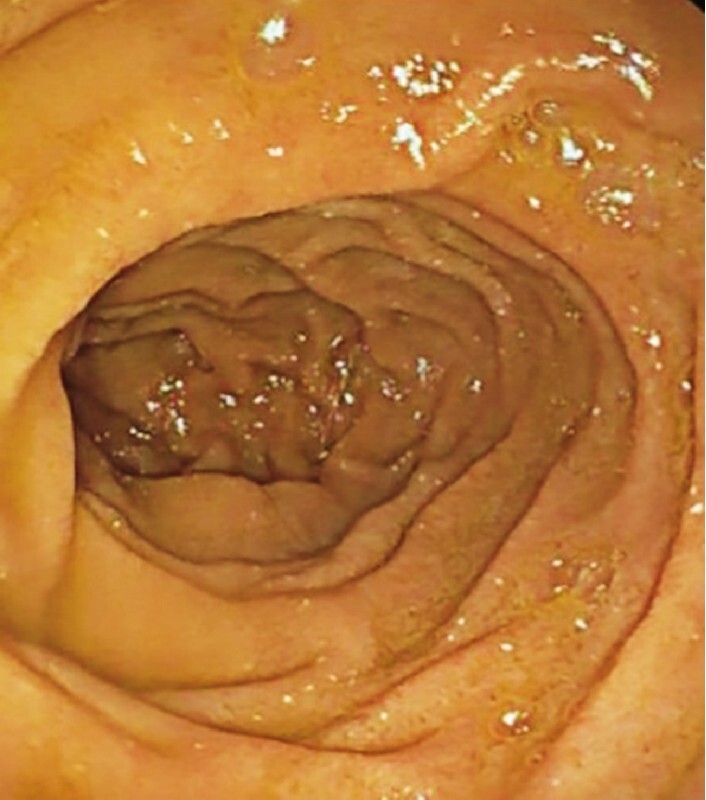
Duodenal view during the first esophagogastroduodenoscopy prior to biopsies being taken.

**Fig. 2:**
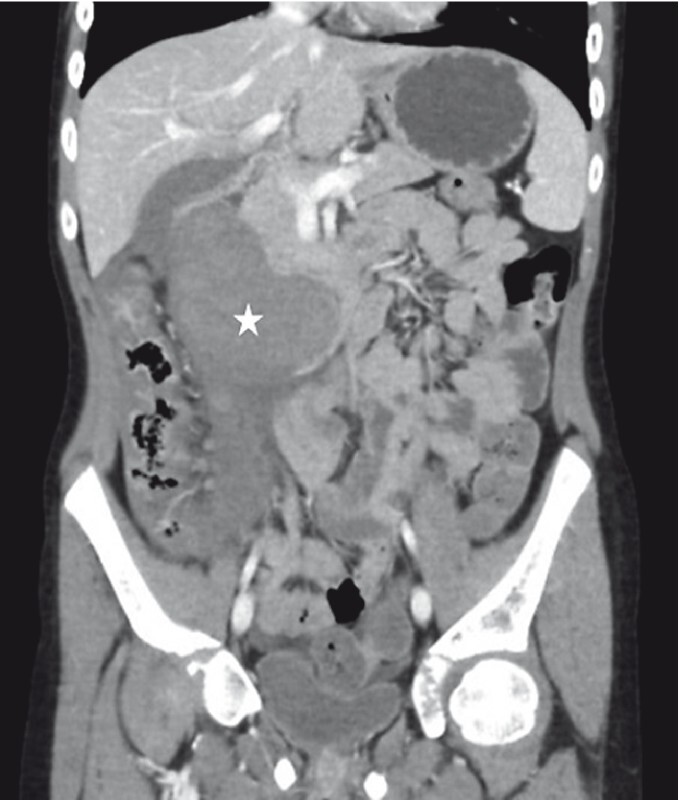
Computed tomography scan image (coronal view) showing a large intramural duodenal hematoma (*).

The patient was treated with total parenteral nutrition, systemic antibiotics, proton pump inhibitors, and nasogastric tube suction. On day 11, a repeat EGD showed a large IDH bulging into the duodenum ( Fig. 3 a ; Video 1 ). An unsuccessful attempt to pass the obstruction with an 8-mm instrument over a guidewire ( Fig. 3 b ) was made. A 5-mm endoscope (SN143POK241; Fujifilm) was however successfully passed through the narrowed lumen at the stenosis, which allowed us to check that the ampulla of Vater was not compressed ( Fig. 3 c ). After this maneuver, the duodenal lumen appeared patent, with an improvement in the degree of obstruction.

**Fig. 3:**
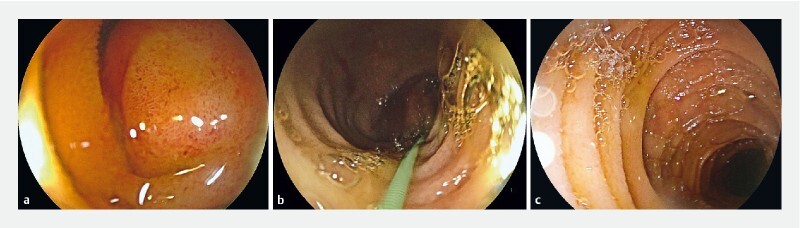
Endoscopic images showing: **a** the intramural duodenal hematoma; **b** a guidewire crossing the duodenal hematoma; **c** the duodenal lumen beyond the hematoma.

**Video 1** Endoscopic and computed tomography views of a huge intramural duodenal hematoma in a 13-year-old girl with undiagnosed platelet dysfunction after diagnostic duodenal biopsies were previously taken during esophagogastroduodenoscopy.


On the 15th postoperative day, the nasogastric tube was removed. The day after its removal, the patient tolerated oral feeding, which was then gradually increased. An ultrasound scan on day 25 showed near-complete resolution of the hematoma and the patient was discharged. Full resorption of the IDH was confirmed on follow-up ultrasound 1 month later.

IDH is a very rare complication after endoscopic biopsies, especially in the pediatric population. Its treatment is still debated, but conservative management should be considered the first choice. Furthermore, an accurate anamnesis is fundamental in the diagnostic assessment in order to prevent serious complications, such as the one described here.

Endoscopy_UCTN_Code_TTT_1AO_2AB

## Notes

Endoscopy E-Videos

https://eref.thieme.de/e-videos
*Endoscopy E-Videos* is an open access online section, reporting on interesting cases and new techniques in gastroenterological endoscopy. All papers include a high quality video and all contributions are freely accessible online. Processing charges apply (currently EUR 375), discounts and wavers acc. to HINARI are available. This section has its own submission website at https://mc.manuscriptcentral.com/e-videos
